# Effect of Income Heterogeneity on Valuation of Mortality Risk in Taiwan: An Application of Unconditional Quantile Regression Method

**DOI:** 10.3390/ijerph16091620

**Published:** 2019-05-09

**Authors:** Je-Liang Liou

**Affiliations:** The Center for Green Economy, Chung-Hua Institution for Economic Research, No. 75 Chang-Hsing Street, Taipei 10672, Taiwan; jlliou@cier.edu.tw; Tel.: +886-2-2735-6006 (ext. 423)

**Keywords:** value of statistical life, income heterogeneity, hedonic wage function, Box-Cox transform, unconditional quantile regression

## Abstract

According to theory and existing empirical results, heterogeneity in personal characteristics, with income variation being one of them, affects the marginal willingness to pay (WTP) for reducing fatal risk. In this study, the effect of income heterogeneity on the value of statistical life (VSL) in Taiwan through unconditional quantile regression analysis using the data collected by the “Manpower Utilization Survey” is investigated. The results of this empirical study show that the hedonic wage function that was constructed using empirical data from Taiwan was in line with the general form of non-linear function rather than the semi-log function that has been often used in previous studies, which should have great impact on the estimation of the VSL. The empirical results also show that the estimated VSL of Taiwanese labor varied with the difference in wages, which needs to be taken into account when discussing the public policies using VSL.

## 1. Introduction

In recent years, with the limited national budget, cost-benefit assessment (CBA) has been adopted to examine the efficiency of public construction plans and management policies, and it is becoming an important reference for making decisions on different plans or policies by public agencies. In general, the construction plans or management policies that were implemented by public agencies involve external influences that are often ignored by the private sector. Among these influencing factors, the changes in the impact of risks of health or death are often the focus of assessment programs in the public sector [1].

The effect on health or death is often measured with changes in risk indicators, but, in the context of assessment of public policies, it is necessary to conduct a monetized evaluation to enable risk indicators to be effectively integrated into the CBA decision analysis framework. From a methodological point of view, the commonly used fatal risk monetization method is the value of statistical life (VSL). According to the basic concept of VSL, there is a willingness to pay (WTP) for the reduction of a certain percentage of fatal risk in the minds of people, and VSL refers to the summing up of the WTPs in the unit of risk [2,3]. VSL plays a very important role in the assessment of public decision-making, especially in applications of environmental protection, public health care, food safety, transportation policy, and so on [4,5,6,7]. Based on past assessment experience, the benefits or costs that are generated by VSL in the aforementioned public construction or management decisions often account for more than 80% of the total benefit or total cost [8]. Therefore, the evaluation and updating of VSL has become an important task in supporting the public sector’s management decisions.

In terms of methodology, VSL assessment can be performed through two evaluation methods. The first is the stated preference approach, in which questionnaire surveys, such as the contingent valuation method (CVM), are adopted to measure the WTP for reducing the fatal risk of the general public and to further to estimate VSL [9,10,11,12,13,14,15,16,17]. The second is the hedonic wage method (HWM), in which the difference in fatal risk behind wage is used to infer the evaluation of fatal risk by wage-earners in different occupations and to further estimate VSL. The cost of the actual operation is rather high since the application of CVM involves the use of a questionnaire. On the contrary, the HWM estimates VSL while using the wage data of the labor market, which are often readily available, thereby resulting in a low operating cost. Thus, HWM is currently the dominant approach in estimating VSL, which has been extensively used [18,19,20,21,22,23,24,25,26,27,28,29]. 

There are various sources of heterogeneity in the estimates of VSL, and income variation is one of them. From a theoretical point of view, because reducing fatal risk is often a normal good, the population segment with high income should also have a high WTP for reducing fatal risk and vice versa [2,8,18,19]. The connotations of income heterogeneity on VSL are various, since the income variation can be defined based on an inter-temporal period, across countries, or within a single group (e.g., labor market), for a specific time point. Therefore, the policy implications of the analysis of income heterogeneity on VSL are also different.

Within the HWM framework, there are two approaches to analyze the income heterogeneity on VSL. The first one is for analysis across studies, and the second one is for within a single sample. In terms of the first approach, a meta-analysis method is primarily used to examine the effect of income heterogeneity on VSL through the use of a large number of existing VSL-related studies [1,2,30]. The analysis that is based on this approach usually has implications for inter-temporal and cross-nations benefit transfers. Applying the meta-analysis method to analyze the income heterogeneity requires sufficient existing studies as its analytical basis, which is a prerequisite of this approach. Therefore, in the absence of adequate literature, it is difficult to investigate the impact of income heterogeneity on VSL through meta-analysis. Besides, since the study of the effect of income heterogeneity on VSL using meta-analysis usually assumes that there are no unobserved differences across observations, consequently, this approach does not reflect the full range of heterogeneity within the sample [31].

Evans and Schaur [18] and Kniesner et al. [19] employed a conditional quantile regression model to conduct empirical analysis on the hedonic wage function, which is the second approach towards examining the impact of income heterogeneity on VSL. Its ability to estimate the VSL values corresponding to different points in the wage distribution characterizes this approach, being able to make up for the limitation of meta-analysis. The results of the above empirical studies supported the conclusion that income heterogeneity exerts a significant impact on VSL. Until now, applying a quantile regression framework to obtain the estimates of VSL is the only approach that can be used to analyze the income heterogeneity on VSL within a single sample [19,31]. Although the conditional quantile regression method that was applied by Evans and Schaur [18] provides a pragmatic approach for understanding the impacts of income heterogeneity to the estimation of VSL, it still has some limitations on policy interpretation. Due to the technical nature of the conditional quantile regression, it can only be used to measure the impacts of fatal risk on a quantile of the wage conditional on specific values of other covariates. As a result, the interpretation of income heterogeneity influence on VSL estimation becomes limited when the fatal risk effects for different conditional quantiles vary. The adoption of an unconditional quantile regression method is necessary in order to overcome the problems of conditional quantile regression that are mentioned above and to provide more interpretable policy insights [32,33,34,35].

In Taiwan, regulatory impact analysis (RIA) for public policy is the current driving direction. Therefore, providing a credible and updated VSL estimation for the public sector is an important task. While reviewing the existing VSL literature in Taiwan, it is found that there is still room for improvement. Prior to 2000, there were several studies, including Wang [36], Hsueh and Wang [37], Liu and Zhan [38], and Liu et al. [39]. However, after 2000, Liu only conducted one empirical study [40], which involved an estimation of VSL from 2002–2006. Without VSL being estimated by up-to-date data, the decision makers cannot know the responsiveness of VSL to income transition in recent years. This is the first inadequacy. Secondly, the effect of income heterogeneity on VSL within a single sample has not been investigated in Taiwan so far. This means that the evaluation of mortality risk reduction for different income groups cannot be measured.

Based on those regards that are mentioned above, this study employed the unconditional quantile regression method to conduct an empirical analysis on the impact of income heterogeneity on VSL in Taiwan to bridge the gap of VSL research in Taiwan, based on HWM. There are two main contributions of this study. First, as far as I know, the VSL estimation results of this study are the newest ones in Taiwan. They can replace the older VSL and be used for RIA and CBA in policy assessment. Second, our results provide more information regarding the distribution of VSL corresponding to different wage levels, thus providing more insights for public decision makers to design policy measures. 

## 2. Research Methods

### 2.1. Application of HWM in VSL

HWM establishes the hedonic wage function through the wage-risk tradeoff relationship, in which the assessments of the changes in fatal risks by job seekers are inferred through the wage differences that correspond to the fatal risks that are faced by employees of different occupations, which are then used as the basis for VSL estimation. In the settings of HWM, it is usually assumed that the wage has a functional relationship between the job and the personal characteristics, i.e., the wage (*W_i_*) of a particular sample *i* can be expressed as a function that various variables, such as personal characteristics (S*_i_*, e.g., socio-economic background features, such as education level, gender, age, work experience, etc.), job characteristics (*N_i_*, e.g., occupation, work location, etc.), fatal risk of the job (*FR_i_*), nonfatal injury risk of the job (*NFR_i_*), etc., affect, as shown in Formula (1).

(1) $$W_{i}=f\left(S_{i},N_{i},FR_{i},NFR_{i}\right)$$

By calculating the use of the empirical data on Formula (1), the coefficient of each characteristic is obtained, which represents the marginal impact of the per-unit change in each characteristic on wage *W_i_,* and that *FR_i_* represents monetized assessment, which corresponds to per-unit change in fatal risk probability, which can be further used to infer VSL.

In terms of the specification of empirical function, following the settings in the linear-log hedonic wage function that has been most commonly used in existing literature, VSL can be calculated while using Formula (2).

(2) $$\mathrm{VSL}\left(FR_{i}\right)=\left[\left(\frac{\partial \hat{lnW_{i}}}{\partial FR_{i}}=\hat{\delta }\times W_{i}\right)\times \mathrm{annual}\;\mathrm{working}\;\mathrm{hours}\times \mathrm{unit}\;\mathrm{of}\;\mathrm{risk}\;\mathrm{probability}\right]$$

In which $\hat{\delta }$ is the estimated coefficient of fatal risk probability *FR_i_*; the annual working hours are generally set to 2000 h (40 h/week, 50 weeks/year); and, the unit of risk probability depends on the unit of probability that is adopted in each specific study. For example, the occupational injuries ratio per thousandth under labor insurance in Taiwan mainly uses an index of one thousand persons (1000%) as the risk measurement unit and, thus here, the unit of risk probability is 1000.

### 2.2. Unconditional Quantile Regression Model with Endogeneity

The quantile regression model is a semi-parametric method, in which the distribution is presented based on actual data, and the estimation of coefficient corresponding to the specific conditional quantile is obtained but it does not require predetermining the form of the distribution function, so it is more flexible in applications of empirical estimation. Another advantage of the quantile regression approach is that, when the samples themselves are asymmetric, outliers less affect it and thus gives rise to a better estimate of the coefficient. As a result, quantile regression has become a popular empirical analysis tool in recent years [41,42].

The quantile regression framework that has been extensively used in the applied economics literature is usually based on the conditional quantile regression model. The conditional quantile regression model can only be used to measure the impacts of major treatment variable, i.e., the fatal risk in this study, on a quantile of the dependent variable, conditional on specific values of other covariates. As a result, the interpretation of treatment variable influences on the dependent variable becomes limited when such influences for different conditional quantiles change [33]. However, decision makers are often interested in the relationship between the major treatment variable and the outcome distribution, unconditional on additional covariates, which can only be measured by the unconditional quantile regression method [32,33,34,35]. 

Based on those methodological regards that are mentioned above, in this study, the concept of traditional HWM was adopted and the hedonic wage function was estimated while using the unconditional quantile regression approach. Formula (3) shows the empirical formula.

(3) $$W_{i}\left(FR_{i}\right)=\alpha_{\theta }+S_{i}^{\prime }\beta_{\theta }+N_{i}^{\prime }\gamma_{\theta }+\delta_{1\theta }\left(FR_{i}\times Age_{i}\right)+\delta_{2\theta }(NFR_{i}\times Age_{i})+\varepsilon_{\theta i}$$

Where in *i* represents different samples; θ represents different conditional quantiles; *W* is a variable measuring the hourly wage and assumes a certain functional relationship with *FR; S* is the vector of variables of personal socio-economic background; *N* is the vector of working environment variables; *FR* is the fatal risk corresponding to each of the different occupations; *NFR* is the non-fatal injuries risk corresponding to each of the different occupations; *Age* is the age of the respondent; β, γ, and δ are coefficients to be estimated; and, ε is the random error term. Of these, the interaction terms between *Age* and *FR* and *NFR* measure the effect of age differences on VSL estimation results, which have already been shown to be significant factors [2,11,18,21,25,31,40].

One fundamental assumption of the quantile regression is that all of the covariates are exogenous. However, there may be some unobservable variables that are correlated with the major treatment variable of fatal risk included in the hedonic wage model, leading to endogeneity bias. The instrumental variable approach (IV) is the main solution for this problem, according to some existing VSL literatures that take the endogeneity issues into account [43,44]. To avoid the possible influence that is caused by endogeneity bias, this study followed the concept of Kniesner et al. [44], using a lagged variable of fatal risk, i.e., *FR*_*t* − 1_, as an instrument to solve for the possible endogeneity of the major treatment variable *FR*. It is important to note that the conventional estimation process of IV, like two-stage least squares (2SLS), is not applicable to unconditional quantile regression framework. Therefore, an advanced estimation process is required. Until now, there are only two approaches that can work in an unconditional regression framework. Frölinch and Melly proposed the first one [34], which employed an instrumental variable to solve the endogeneity of a binary treatment variable. Another one, which is an estimation process based on generalized quantile regression estimator (GQR) was developed by Powell [35], which does not only apply to the endogeneity of the binary treatment variable, but also to the continuous one. Since the major treatment variable in this study (*FR*) is a continuous variable, the GQR-IV estimation process was adopted to solve the endogeneity bias.

The characteristics of quantile regression are that under different quantiles, the marginal effect of each characteristic’s variable on wage can be estimated. Let the hedonic wage function assume the semi-log function form; then, based on this characteristic of quantile regression, the ${\mathrm{VSL}}_{\theta }\;$corresponding to different wage quantile intervals (θ) can be calculated while using Formula (4):

(4) $${\mathrm{VSL}}_{\theta }\left(FR_{i,iv}\right)=\left[\left(\frac{\hat{\partial lnW_{i}}}{\partial FR_{i,iv}}={\hat{\delta }}_{\theta }\times Age_{i}\times W_{i}\right)\times 2000\times 1000\right]$$

Where, $FR_{iv}$ is the instrumental variable in the GQR-IV estimation process.

### 2.3. Choice of Empirical Function Form: Box-Cox Transform 

In studies involving VSL, the choice of the form of hedonic wage function exerts a certain impact on the estimation of VSL. To avoid the issues that are associated with the subjective choice of the function form, Moore and Viscusi [45] recommended the use of Box–Cox transform, in which a statistical test is used to determine the form of hedonic wage function. The study found that the semi-log function was more fitted to the empirical data from the United States (US). Due to this characteristic, subsequent studies of VSL using US empirical data also adopted the setting of semi-log function.

The empirical data from Taiwan may be different from those from the US that have been used by Moore and Viscusi [45] in various aspects, such as structure and variable characteristics, so directly choosing the semi-log function form that is based on the empirical study is inappropriate. To avoid the influence of the subjective choice of empirical function form, this study adopted the Box–Cox method to perform the estimation in combination with Formula (5) and determined the optimal empirical function form based on the test result.

(5) $$\frac{W_{i}^{\lambda }-1}{\lambda }=\alpha +S_{i}^{\prime }\beta +N_{i}^{\prime }\gamma +\delta_{1}\left(FR_{i,iv}\times Age_{i}\right)+\delta_{2}\left(NFR_{i}\times Age_{i}\right)+\varepsilon_{i}$$

Where in λ is the characteristic’s coefficient in the Box–Cox transform, and the optimal value of λ can be obtained through estimation using Formula (5), from which the optimal hedonic wage function form for the data from Taiwan can be inferred. The forms of the function can vary as λ changes, as shown in Formula (6).

(6) $$\frac{W^{\lambda }-1}{\lambda }=\{\begin{array}{c} W-1,\;if\;\lambda =1\\ \ln\left(W\right),\;if\;\lambda =0\\ 1-\frac{1}{W},\;if\;\lambda =-1 \end{array}$$

## 3. Empirical Data Source and Processing

### 3.1. Manpower Utilization Survey

In this study, the data from the “Manpower Utilization Survey”, which is annually executed by the Directorate-General of Budget, Accounting and Statistics, Executive Yuan, Taiwan, were used in the empirical estimation of the hedonic wage function. According to the instruction of the Directorate-General of Budget, Accounting and Statistics, Executive Yuan [46], the objects of the “Manpower Utilization Survey” are citizens of Taiwan from ordinary households and common business households residing in Taiwan who are aged 15 and above and engage in economic activities; households are annually surveyed through interviews with respondents by designated personnel.

The survey questionnaire contains three major parts. In the first part, items, such as the monthly income, regular weekly working hours, employment duration of current job, the number of job changes, the workplace location and title of last job, reasons for leaving last job, the method of obtaining current job, and the plan of changing job or adding more jobs are included to understand the utilization of manpower and employees’ job changes. In the second part, items, such as sought-after jobs and the expected benefits of the unemployed, the acceptance of grassroots jobs with irregular working hours and manufacturing or construction sites, employment opportunities during job hunting and the reasons for not being employed, and the source of living expenses during job hunting are included. In the third part, the items are designed for the currently unemployed, such as information regarding last job and reasons for stopping last job, information on job hunting and reasons for stopping job-hunting, employment willingness and expected salary and benefits, etc., and the age information for the respondent’s children is also included to understand the influence of children in the household on the labor participation of the respondent.

The information that was collected in the “Manpower Utilization Survey” contains the personal characteristics and working characteristics variables that are needed for estimating hedonic wage function and it represents the most complete survey data of labor force employment in Taiwan. Thus, it is suitable for the empirical estimation of hedonic wage function in Taiwan. The 2014 survey data were chosen for the subsequent empirical analysis in this study.

### 3.2. Source and Processing of Fatal Risk and Non-Fatal Injuries Risk Variables

Two risk variables, i.e., fatal risk (*FR*) and non-fatal injuries risk (*NFR*) in different occupations, were measured using the items that were included in the statistical data in “Occupational injuries ratio per thousandth under labor insurance” from the Ministry of Labor [47]. In addition to the use of the occupational fatality rate that corresponds to each of different occupations as the measurement of the fatal risk variable, two additional indicators, i.e., annual occupational injury or sickness ratio per thousandth and disability ratio per thousandth, were used, and the sum of the two was used as the measurement of the non-fatal injuries risk (*NFR*) variable.

### 3.3. Sample Processing

To focus on the research topics of this study, the data with missing information were excluded and further processed, as follows:

- based on the general life cycle of labor, those who are aged below 20 years or over 65 years were excluded;
- those who receive monthly wages were used as analysis objects;
- full-time employees were used as analysis objects, and part-time ones were excluded;
- those who are self-employed, employers, and unpaid homemakers were excluded;
- those who earn a wage below the minimum wage according to Taiwan’s official standard (with a monthly salary of less than $625.4, or an hourly rate of $3.79; the 2014 average exchange rate of NT$ dollar to US dollar: 30.38:1) were excluded; and,
- those who have missing information on monthly salary and working hours were excluded.

After screening and processing according to the above criteria, a total of 3,974 samples were obtained for subsequent empirical analysis.

The empirical variables that were used in this study can be divided into three broad categories: i.e., risk variables (fatal risk *FR*, nonfatal injury risk *NFR*), personal characteristics variables (S), and working environment variables (N). Table 1 shows the definition and descriptive statistics of each variable.

## 4. Empirical Results and Discussions

### 4.1. Choice of Function Form: Box-Cox Estimation Result

The STATA software was adopted to estimate the Box­–Cox transform as the basis for setting the form of hedonic wage function. The estimation result of the Box–Cox transform shows that the transform coefficient (λ) is −0.7291 (Table 2), which is significant at the 1% level and different from 0, which indicates that the hedonic wage function with the empirical data from Taiwan does not show the semi-log relationship, but a more ordinary nonlinear relationship. Aside from that, the collinearity diagnosis via variance inflation factor (VIF) was implemented. The mean value of VIF is about 5.06 < 10, which indicates that the collinearity problem is not serious in this study.

Thus, based on the estimation result of coefficient value (λ) and Formula (7), the hourly wage after nonlinear conversion was redefined and used as the dependent variable to perform a subsequent empirical estimation on the hedonic wage function.

(7) $$BC\_hour\_wage=\frac{W_{i}^{\lambda }-1}{\lambda }=\frac{W_{i}^{-0.7291}-1}{-0.7291}$$

### 4.2. Estimation Result of Hedonic Wage Function

To estimate the VSL at different income intervals, this study used the quantile regression model to estimate the hedonic wage function under nine quantiles, i.e., the 10th percentile (θ = 0.1), the 20th percentile (θ = 0.20), the 30th percentile (θ = 0.3), the 40th percentile (θ = 0.4), the 50th percentile (θ = 0.5), the 60th percentile (θ = 0.6), the 70th percentile (θ = 0.70), the 80th percentile (θ = 0.8), and the 90th percentile (θ = 0.9). Moreover, the 2SLS was also employed, which has been frequently used in previous studies, to estimate, and the estimation result was compared with that obtained while using quantile regression estimation. In the estimations, virtual variables that have been excluded to be references, i.e., “workplace location is outside Taiwan’s five municipalities” *(area_6)*, “having an education level of Ph.D.” *(Edu8)*, “the number of employees of the company of employment is over 500” *(Indus_size7*), and “the occupational category is mechanical equipment and assembly operators” (*Occu9*), were used as the comparison baselines. The “genqreg” STATA code that was developed by Powell [35] was used to estimate the unconditional quantile regression model under endogeneity in this study. Table 3 shows the coefficient estimation results of each model.

The results in Table 3 show that the estimates of coefficients through the unconditional quantile regression model and the conventional 2SLS estimation were consistent in signs. In terms of the interaction term of occupational fatal risk and age *(FR*×*Age)*, which is the most important for VSL estimation, the estimated coefficients were all statistically significant and varied with differences in income levels, which confirms the presence of income heterogeneity. The comparison on the estimation results of 2SLS and unconditional quantile regression show that the estimated coefficients by unconditional quantile regression at all percentiles were all higher than those by 2SLS, indicating that, in addition to its inability to reasonably present the fatal risk evaluations under different income levels, the traditional 2SLS underestimated VSL. Furthermore, the signs of the interaction terms of fatal risk and age by different models were all positive, which indicates that the evaluation of fatal risk increased as age increased. This result also indicated that, in terms of the empirical data from Taiwan, the higher the age, the higher the VSL that was inferred, which is consistent with the result of Smith et al. [21].

### 4.3. VSL Estimation under Different Income Levels 

In this study, the hedonic wage function, which was constructed using empirical data from Taiwan, was judged as a general non-linear function through a Box–Cox transform test, and the marginal effect of per-unit wage change on income can be derived through Formula (8). On this basis, in combination with the estimation on the coefficient of the interaction term of fatal risk and age in each quantile regression model, the mean VSL value corresponding to each income quantile can be calculated through Formula (9); Table 4 shows the result of the calculation.

(8) $$\partial \left(\frac{W_{i}^{\lambda }-1}{\lambda }\right)/\partial FR_{i}={\hat{\delta }}_{1}\times Age_{i}⟹\partial W_{i}/\partial FR_{i}=\left({\hat{\delta }}_{1}\times Age_{i}\times W_{i}^{1-\lambda }\right)$$

(9) $$\bar{{\mathrm{VSL}}_{\theta }}\left(FR\right)=\left[\left({\hat{\delta }}_{\theta }\times \bar{\mathrm{Age}}\times \bar{W^{1-\lambda }}\right)\times 2000\times 1000\right],\;\lambda =-0.7291$$

The results in Table 4 show that first the VSL values corresponding to different income levels were significantly different, which indicates that income heterogeneity is indeed present in the estimated VSL values of Taiwan. The comparison of VSL values at the 50th percentile (i.e., median) by 2SLS and quantile regression indicates that the mean VSL value that was estimated through 2SLS was $6.05 million, which is lower than that estimated through quantile regression at the 50th percentile ($9.74 million). Using the meta-analysis method, the United States Environmental Protection Agency [1] estimated the mean VSL value at $10.3 million (in 2013 $). When compared with that of the US, the mean estimated VSL value of Taiwan was still lower.

In terms of the VSL values that were estimated under different income levels, except for the VSL value that was estimated at the 10th percentile for wage, the VSL values estimated at the other percentiles increased as wage generally increased, which is consistent with the trend revealed in previous studies. A comparison between wage at the 80th percentile and that at the 50th percentile, which corresponds to a monthly wage difference of approximately 45%, shows that their estimated VSL values differed by approximately 161%. The estimated VSL value at the 90th percentile increased by approximately 82% when compared with that at the 50th percentile, while the monthly wage difference at the two percentiles was approximately 228%. This result indicates, that in the high-wage quantile intervals, the gain to VSL from per-unit wage was also high.

Table 4 shows that the estimated VSL value at the 10th percentile was significantly higher than that at the 20th or 30th percentile, which seems to be inconsistent with the inference that, the higher the wage, the higher the VSL. Further investigation revealed that this inconsistency may stem from the impacts of other types of heterogeneity other than income heterogeneity. Previous studies indicated that, in addition to income heterogeneity, age heterogeneity is also a factor that exerts a significant impact on VSL [2,11,18,21,25,31,40]. To further grasp the effect of age heterogeneity on the estimated VSL value of Taiwan, Formula (10) is used to estimate the VLS for each age group at each percentile, and Table 5 shows the calculation results.

(10) $${\mathrm{VSL}}_{\theta ,Age_{j}}\left(FR\right)=\left[\left({\hat{\delta }}_{\theta }\times Age_{j}\times \bar{W^{1-\lambda }}\right)\times 2000\times 1000\right],\;Age_{j}=20,30,40,50,60.$$

The conclusions on the effect of age heterogeneity on VSL in previous studies are inconsistent. Some found that, the higher the age, the lower the VSL, while others drew the opposite conclusion. According to the calculation results shown in Table 5, in the empirical data of Taiwan, age had a positive impact on VSL, i.e., the higher the age, the higher the VSL, indicating that, even under the same wage interval, the distribution of age groups will affect the interpretation of VSL.

To examine the raw data and found that in 465 samples, the hourly wage was below $4.46 (corresponding to the 10th percentile of income level), and those that were aged over 30 years accounted for 70% of the total. As VSL rises with age increase, in the low−wage intervals where high-aged laborers compose a high proportion, the estimated VSL was also higher.

Only a few previous studies have simultaneously addressed the effects of income heterogeneity and age heterogeneity on VSL. Evans & Schaur [18] also employed quantile regression to simultaneously examine the influences of income heterogeneity and age heterogeneity on VSL in the US and found that, under the 50th percentile for wage (equivalent to an hourly wage of $13.07), the VSL of those that were aged 50 years was $65.59 million. In the case of Taiwan, the hourly wage of $13.07 represented the 90th percentile for wage in Taiwan, and the estimated VSL of those aged 50 years was $40.66 million, which is much lower than that of the US.

## 5. Conclusions

In this study, the effect of income heterogeneity on VSL was examined through the quantile regression method while using 2014 “Manpower Utilization Survey” data, and the results of this empirical study show that first, the more appropriate setting for constructing the hedonic wage function using the empirical data from Taiwan is the general non-linear form rather than the semi-log form that has been commonly used in previous studies. When the semi-log form is used to estimate Taiwan’s VSL, it is prone to generating errors.

Second, the estimated VSL values of Taiwan significantly fluctuated with income differences, which is consistent with the results of previous studies. This finding also indicates that, income heterogeneity must be taken into account when estimating Taiwan’s VSL. Moreover, in this study, the effect of age heterogeneity was also simultaneously considered, and the comparisons on VSL values were made in combination with age heterogeneity under different income levels. Overall, the higher the wage, the higher the age, and the higher the WTP for reducing fatal risk.

In terms of policy implications, because the combination of wage and age was used to estimate VSL in this study, the findings can be applied in more detailed cost−benefit analyses in case studies, thereby improving the quality of public decision−making. For example, assessing the health benefits of implementing control measures for air pollution has been a high-profile issue in recent years. These assessments help the government to decide the priority order of budget plan under budgetary constraints. Among the health benefit metrics in such assessments, VSL is the primary one usually adopted to assess the benefits of fatal risk reduction due to the implementation of control measures. Since the income and age heterogeneity significantly affect VSL, when combined with the distribution status of different income and age populations in each region and diffusion simulation of air pollution, the estimated VSL in this study can be used to convert the air pollution health risk map into a health cost map. The use of the health cost map of air pollution will certainly help policymakers to formulate the control strategies based on cost−benefit principles, which increases the efficiency of public budget usage.

Lastly, as the empirical data of only a single year were used in this study, the effect of time dimension on VSL is lacking, which is one limitation of this study. In spite of that, this study demonstrates the unconditional quantile regression model is a pragmatic method in VSL estimation and it has obtained meaningful results, which provides a good reference for subsequent research. Therefore, an investigation on the cross-year VSL changes while using the empirical estimation process established in this study can be conducted in follow-up studies to gain more policy insights.

## Figures and Tables

**Table 1:** Empirical variables and Summary statistics.

Variable	Definition	Mean	Standard Deviation
*hour_wage*	Hourly wage rate(2014 NT$)	220.3872	103.75
*FR_t_*	Fatalities per 1000 workers in the individual’s industry (t = 2014)	0.0259	0.0407
*FR*_*t* − 1_	Fatalities per 1000 workers in the individual’s industry (t − 1 = 2013)	0.0123	0.0121
*NFR*	Injury per 1000 workers in the individual’s industry	3.1558	2.4898
*Area_1*	Dummy variable that equals 1 if individual’s location of workplace is in Taipei City	0.0722	0.2589
*Area_2*	Dummy variable that equals 1 if individual’s location of workplace is in New Taipei City	0.1062	0.3081
*Area_3*	Dummy variable that equals 1 if individual’s location of workplace is in Taichung City	0.1256	0.3314
*Area_4*	Dummy variable that equals 1 if individual’s location of workplace is in Tainan City	0.0833	0.2764
*Area_5*	Dummy variable that equals 1 if individual’s location of workplace is in Kaohsiung City	0.1233	0.3288
*Area_6*	Dummy variable that equals 1 if individual’s location of workplace is outside of the five municipalities in Taiwan	0.4894	0.5000
*familysize*	Number of family members over 15 years old	3.6228	1.5132
*Age*	Individual’s age in years	39.2285	10.0255
*Sex*	Dummy variable indicating individual is male	0.5528	0.4973
*Exp*	Total number of years worked	8.3132	7.2991
*Edu1*	Dummy variable that equals 1 if individual’s education attainment is primary school	0.0216	0.1455
*Edu2*	Dummy variable that equals 1 if individual’s education attainment is junior high school	0.0893	0.2853
*Edu3*	Dummy variable that equals 1 if individual’s education attainment is senior high school	0.2343	0.4236
*Edu4*	Dummy variable that equals 1 if individual’s education attainment is vocational high school	0.0941	0.2920
*Edu5*	Dummy variable that equals 1 if individual’s education attainment is junior college	0.1545	0.3615
*Edu6*	Dummy variable that equals 1 if individual’s education attainment is university	0.3261	0.4689
*Edu7*	Dummy variable that equals 1 if individual’s education attainment is master	0.0730	0.2601
*Edu8*	Dummy variable that equals 1 if individual’s education attainment is Ph.D	0.0065	0.0806
*Marital*	Dummy variable that equals 1 if individual is single without spouse	0.3958	0.4891
*Indus_size1*	Dummy variable that equals 1 if number of employees of company is 2-9 persons	0.1993	0.3995
*Indus_size2*	Dummy variable that equals 1 if number of employees of company is 10-29 persons	0.2028	0.4021
*Indus_size3*	Dummy variable that equals 1 if number of employees of company is 30-49 persons	0.1002	0.3002
*Indus_size4*	Dummy variable that equals 1 if number of employees of company is 50-99 persons	0.0888	0.2845
*Indus_size5*	Dummy variable that equals 1 if number of employees of company is 100-199 persons	0.0924	0.2896
*Indus_size6*	Dummy variable that equals 1 if number of employees of company is 200-499 persons	0.0541	0.2262
*Indus_size7*	Dummy variable that equals 1 if number of employees of company is above 500 persons	0.1090	0.3116
*Public_sector*	Dummy variable that equals 1 if individual worked in public sector	0.1535	0.3605
*Occu1*	Dummy variable that equals 1 if individual’s occupation belongs senior officials and chief executives	0.0345	0.1825
*Occu2*	Dummy variable that equals 1 if individual’s occupation belongs technicians and associate professionals	0.2116	0.4085
*Occu3*	Dummy variable that equals 1 if individual’s occupation belongs craft and related trades workers	0.1251	0.3308
*Occu4*	Dummy variable that equals 1 if individual’s occupation belongs clerical support workers	0.1490	0.3561
*Occu5*	Dummy variable that equals 1 if individual’s occupation belongs service workers and sales	0.1095	0.3123
*Occu6*	Dummy variable that equals 1 if individual’s occupation belongs elementary labourers	0.0365	0.1875
*Occu7*	Dummy variable that equals 1 if individual’s occupation belongs professionals	0.1590	0.3658
*Occu8*	Dummy variable that equals 1 if individual’s occupation belongs skilled agricultural, forestry and fishery Workers	0.0025	0.0501
*Occu9*	Dummy variable that equals 1 if individual’s occupation belongs stationary plant and machine operators	0.1724	0.3777
Number of Observations: 3974			

Note: “*Exp”*, “*Edu”*, “*Indus_size”*, and “*Occu”* are abbreviations of “experience”, “education”, “industry size”, and “occupation”, respectively.

**Table 2:** Estimation result of Box–Cox transformation.

Variable	Estimated Coefficient	Standard Errors	95% Confidence Interval	
λ	−0.7291 ***	822.91	−0.7987	−0.6596
$\mathrm{LR}\;\chi^{2}\left(34\right)$ = 3343.98 ***				

Note: *** indicates significant at 1% level.

**Table 3:** Empirical results from two-stage least squares (2SLS) and unconditional quantile hedonic wage regressions (dependent variable: BC_hour_wage.).

Variable	Unconditional Quantile Regression									2SLS
	10%	20%	30%	40%	50%	60%	70%	80%	90%	
$FR_{iv}\times Age$	0.000277 *** (1.11 × 10^−5^)	0.000221 *** (1.31 × 10^−5^)	0.000231 *** (4.50 × 10^−5^)	0.000285 *** (2.44 × 10^−5^)	0.000303 *** (3.34 × 10^−5^)	0.000275 *** (2.94 × 10^−5^)	0.000366 *** (2.52 × 10^−5^)	0.000791 *** (1.35 × 10^−5^)	0.000991 *** (8.70 × 10^−6^)	0.000188 *** (1.38 × 10^−5^)
NFR×Age	0.0000001 *** (2.0 × 10^−7^)	0.000001 *** (2.40 × 10^−7^)	0.000001 (6.80 × 10^−7^)	−0.000001 (4.10 × 10^−7^)	−0.000002 *** (4.90 × 10^−7^)	−0.000002 *** (3.73 × 10^−6^)	−0.000003 *** (4.30 × 10^−7^)	−0.000008 *** (2.30 × 10^−7^)	−0.000012 *** (1.60 × 10^−7^)	−0.000001 (1.80 × 10^−6^)
*Area_1*	0.001378 *** (4.57 × 10^−5^)	0.001430 *** (6.35 × 10^−5^)	0.001080 *** (8.64 × 10^−5^)	0.001471 *** (1.38 × 10^−4^)	0.001823 *** (1.47 × 10^−5^)	0.001808 *** (1.0 × 10^−4^)	0.001974 *** (1.46 × 10^−4^)	0.002127 *** (8.36 × 10^−5^)	0.002297 *** (6.16 × 10^−5^)	0.00175 *** (3.05 × 10^−4^)
*Area_2*	0.000850 *** (4.92 × 10^−5^)	0.000850 *** (7.0 × 10^−5^)	0.000565 *** (7.13 × 10^−5^)	0.000753 *** (9.50 × 10^−5^)	0.000494 *** (1.26 × 10^−4^)	0.000327 *** (9.59 × 10^−5^)	0.000376 *** (1.39 × 10^−4^)	0.000218 *** (8.31 × 10^−5^)	0.000196 *** (2.46 × 10^−5^)	0.00047 * (2.58 × 10^−4^)
*Area_3*	0.000594 *** (4.0 × 10^−5^)	−0.000022 6.64 × 10^−5^)	−0.000248 ** (7.63 × 10^−5^)	−0.000287 ** (1.22 × 10^−4^)	−0.000272 ** (1.26 × 10^−4^)	−0.000277 *** (1.21 × 10^−4^)	−0.000119 (9.57 × 10^−5^)	−0.000078 (9.49 × 10^−5^)	0.000207 *** (5.78 × 10^−5^)	−0.0000854 (2.42 × 10^−4^)
*Area_4*	−0.000703 ** (4.95 × 10^−5^)	−0.001029 *** (7.11 × 10^−5^)	−0.001285 *** (1.17 × 10^−4^)	−0.001332 *** (1.02 × 10^−4^)	−0.001369 *** (1.67 × 10^−4^)	−0.001322 *** (1.16 × 10^−4^)	−0.001414 *** (1.23 × 10^−4^)	−0.001274 *** (7.26 × 10^−5^)	−0.001035 *** (7.71 × 10^−5^)	−0.0012083 *** (2.83 × 10^−4^)
*Area_5*	−0.000919 *** (4.55 × 10^−5^)	−0.001208 *** (6.13 × 10^−5^)	−0.001343 *** (7.93 × 10^−5^)	−0.001208 *** (7.64 × 10^−5^)	−0.001180 *** (1.01 × 10^−4^)	−0.001268 *** (8.80× 10^−4^)	−0.001001 *** (9.40 × 10^−5^)	−0.000949 *** (1.05 × 10^−4^)	−0.000562 *** (6.27 × 10^−5^)	−0.00113 *** (2.42 × 10^−4^)
*familysize*	−0.0000003 (8.63 × 10^−7^)	−0.000031 * (1.49 × 10^−5^)	−0.000076 *** (2.0 × 10^−5^)	−0.000118 *** (1.91 × 10^−5^)	−0.000149 *** (2.27 × 10^−5^)	−0.000136 *** (2.33 × 10^−5^)	−0.000150 *** (2.59 × 10^−5^)	−0.000193 *** (2.01 × 10^−5^)	−0.000115 ** (5.3 × 10^−6^)	−0.0001283 ** (5.10 × 10^−5^)
*Age*	0.000019 *** (1.55 × 10^−7^)	0.0000041 (4.11 × 10^−6^)	0.000026 *** (4.0 × 10^−6^)	0.00004 *** (6.55 × 10^−6^)	0.000066 *** (5.13 × 10^−6^)	0.000070 *** (5.55 × 10^−6^)	0.000097 *** (4.42 × 10^−6^)	0.000107 *** (3.56 × 10^−6^)	0.000126 *** (1.69 × 10^−6^)	0.0000562 *** (1.19 × 10^−5^)
*Sex*	0.002458 *** (2.62 × 10^−5^)	0.002943 *** (4.77 × 10^−5^)	0.003105 *** (5.95 × 10^−5^)	0.003257 *** (6.64 × 10^−5^)	0.003234 *** (1.11× 10^−4^)	0.003384 *** (6.93 × 10^−5^)	0.003454 *** (5.92 × 10^−5^)	0.003204 *** (5.70 × 10^−5^)	0.003479 *** (2.84 × 10^−5^)	0.0031233 *** (1.66 × 10^−4^)
*Exp*	0.000211 *** (1.59 × 10^−7^)	0.000228 *** (3.78 × 10^−6^)	0.000216 *** (5.0 × 10^−6^)	0.000216 *** (5.02 × 10^−6^)	0.000185 *** (5.74 × 10^−6^)	0.000172 *** (4.57 × 10^−6^)	0.000158 *** (5.89 × 10^−6^)	0.000156 *** (3.90 × 10^−6^)	0.000114 *** (2.70 × 10^−6^)	0.0001884 *** (1.34 × 10^−5^)
*Edu1*	−0.008922 *** (1.67 × 10^−4^)	−0.008084 *** (3.64 × 10^−4^)	−0.008208 *** (4.93 × 10^−4^)	−0.007900 *** (6.36 × 10^−4^)	−0.008033 *** (7.89 × 10^−4^)	−0.008181 *** (9.48 × 10^−4^)	−0.009021 *** (4.02 × 10^−4^)	−0.008283 *** (3.18 × 10^−4^)	−0.004672 *** (2.28 × 10^−4^)	−0.0083917 *** (1.06 × 10^−4^)
*Edu2*	−0.008049 *** (1.91 × 10^−4^)	−0.007079 *** (2.55 × 10^−4^)	−0.007793 *** (4.75 × 10^−4^)	−0.007421 *** (6.68 × 10^−4^)	−0.00711 *** (7.52 × 10^−4^)	−0.007097 *** (8.25 × 10^−4^)	−0.007838 *** (4.96 × 10^−4^)	−0.006572 *** (2.09 × 10^−4^)	−0.004177 *** (1.63 × 10^−4^)	−0.0073933 *** (9.58 × 10^−4^)
*Edu3*	−0.007230 *** (1.90 × 10^−4^)	−0.006746 *** (2.80 × 10^−4^)	−0.007205 *** (4.26 × 10^−5^)	−0.006847 *** (6.69 × 10^−4^)	−0.006373 *** (6.70 × 10^−4^)	−0.006596 *** (8.62 × 10^−4^)	−0.007364 *** (4.64 × 10^−4^)	−0.00631 *** (2.32 × 10^−4^)	−0.004205 *** (1.62 × 10^−4^)	−0.0068345 *** (9.26 × 10^−4^)
*Edu4*	−0.007308 *** (1.55 × 10^−4^)	−0.006658 *** (3.13 × 10^−4^)	−0.007051 *** (4.86 × 10^−4^)	−0.006788 *** (6.60 × 10^−4^)	−0.005936 *** (7.31 × 10^−4^)	−0.005825 *** (8.33 × 10^−4^)	−0.006766 *** (4.50 × 10^−4^)	−0.005661 *** (2.22 × 10^−4^)	−0.004070 *** (1.39 × 10^−4^)	−0.0065956 *** (9.48 × 10^−4^)
*Edu5*	−0.005864 *** (1.77 × 10^−4^)	−0.005351 *** (2.78 × 10^−4^)	−0.005671 *** (4.65 × 10^−5^)	−0.005323 *** (6.76 × 10^−4^)	−0.004759 *** (6.86 × 10^−4^)	−0.005066 *** (8.16 × 10^−4^)	−0.005828 *** (4.46 × 10^−4^)	−0.005001 *** (2.23 × 10^−4^)	−0.003230 *** (1.38 × 10^−4^)	−0.0054207 *** (9.25 × 10^−4^)
*Edu6*	−0.005166 *** (1.67 × 10^−4^)	−0.004439 *** (3.07 × 10^−4^)	−0.004708 *** (4.25 × 10^−4^)	−0.004263 *** (6.65 × 10^−4^)	−0.003408 *** (6.54 × 10^−4^)	−0.003577 *** (8.59 × 10^−4^)	−0.004374 *** (4.61 × 10^−4^)	−0.003503 *** (2.24 × 10^−4^)	−0.001749 *** (1.4 × 10^−4^)	−0.0040912 *** (9.14 × 10^−4^)
*Edu7*	−0.001711 ** (1.67 × 10^−4^)	−0.001152 *** (3.21 × 10^−4^)	−0.001849 *** (4.03 × 10^−4^)	−0.001843 *** (6.61 × 10^−4^)	−0.001213 * (6.66 × 10^−4^)	−0.001511 * (8.33 × 10^−4^)	−0.002622 *** (4.65 × 10^−4^)	−0.001796 *** (2.62 × 10^−4^)	−0.000885 *** (1.65 × 10^−5^)	−0.001758 *** (9.40 × 10^−4^)
*Marital*	−0.000740 *** (1.80 × 10^−5^)	−0.00095 *** (4.48 × 10^−5^)	−0.000869 *** (8.16 × 10^−5^)	−0.000682 *** (8.36 × 10^−5^)	−0.000710 *** (8.56 × 10^−5^)	−0.000648 *** (8.41 × 10^−5^)	−0.000406 *** (8.74 × 10^−5^)	−0.000465 *** (4.90 × 10^−5^)	−0.000640 *** (4.32 × 10^−5^)	−0.0008086 *** (1.90 × 10^−4^)
*Indus_size1*	−0.002298 *** (7.15 × 10^−5^)	−0.002277 *** (8.11 × 10^−5^)	−0.002528 *** (1.05 × 10^−4^)	−0.002371 *** (1.38 × 10^−4^)	−0.002165 *** (1.06 × 10^−4^)	−0.002036 *** (1.15 × 10^−4^)	−0.002392 *** (1.17 × 10^−4^)	−0.002416 *** (1.23 × 10^−4^)	−0.002612 *** (4.41 × 10^−5^)	−0.0021572 *** (3.08 × 10^−4^)
*Indus_size2*	−0.001984 *** (7.15 × 10^−5^)	−0.002076 *** (1.02 × 10^−4^)	−0.002288 *** (1.19 × 10^−4^)	−0.002208 *** (1.44 × 10^−4^)	−0.002018 *** (9.82 × 10^−5^)	−0.001739 *** (1.06 × 10^−4^)	−0.001908 *** (1.09 × 10^−4^)	−0.001936 *** (1.45 × 10^−4^)	−0.002181 *** (4.93 × 10^−5^)	−0.0018518 *** (2.95 × 10^−4^)
*Indus_size3*	−0.001353 *** (7.18 × 10^−5^)	−0.00166 *** (6.19 × 10^−5^)	−0.001971 *** (1.29 × 10^−4^)	−0.002033 *** (1.65 × 10^−5^)	−0.001785 *** (1.27 × 10^−4^)	−0.001756 *** (1.44 × 10^−4^)	−0.002119 *** (1.38 × 10^−4^)	−0.001731 *** (1.23 × 10^−4^)	−0.001518 *** (4.44 × 10^−5^)	−0.0015528 *** (3.38 × 10^−4^)
*Indus_size4*	−0.000970 *** (5.82 × 10^−5^)	−0.000915 *** (7.63 × 10^−5^)	−0.001368 *** (1.55 × 10^−4^)	−0.001396 *** (1.40 × 10^−4^)	−0.001342 *** (1.35 × 10^−4^)	−0.001354 *** (1.71 × 10^−4^)	−0.00139 *** (9.54 × 10^−5^)	−0.001317 *** (1.32 × 10^−4^)	−0.001717 *** (4.37 × 10^−5^)	−0.0011651 *** (3.44 × 10^−4^)
*Indus_size5*	−0.001345 *** (4.46 × 10^−5^)	−0.000763 *** (1.03 × 10^−5^)	−0.000832 *** (7.45 × 10^−5^)	−0.000776 *** (1.10 × 10^−4^)	−0.000476 *** (1.19 × 10^−4^)	−0.000787 *** (1.17 × 10^−4^)	−0.000621 *** (1.46 × 10^−4^)	−0.000737 *** (1.44 × 10^−4^)	−0.00070 *** (3.46 × 10^−5^)	−0.0006715 ** (3.38× 10^−4^)
*Indus_size6*	−0.000284 ** (5.35 × 10^−5^)	−0.000607 *** (7.39 × 10^−5^)	−0.000810 *** (1.32 × 10^−4^)	−0.000828 *** (1.60 × 10^−4^)	−0.000491 *** (1.52 × 10^−4^)	−0.000659 *** (1.57 × 10^−4^)	−0.000481 *** (1.61 × 10^−4^)	−0.000465 *** (1.63 × 10^−4^)	−0.000059 (8.80 × 10^−5^)	−0.0005252 (3.95 × 10^−4^)
*Public_sector*	0.001471 *** (5.69 × 10^−5^)	0.002588 *** (8.02 × 10^−5^)	0.002554 *** (1.41 × 10^−4^)	0.002786 *** (1.28 × 10^−4^)	0.002976 *** (1.39 × 10^−4^)	0.002891 *** (8.36 × 10^−5^)	0.002663 *** (1.37 × 10^−4^)	0.0018900 *** (1.25 × 10^−4^)	0.001453 *** (4.94 × 10^−5^)	0.0024619 *** (3.37 × 10^−4^)
*Occu1*	0.010617 *** (8.41 × 10^−5^)	0.009971 *** (1.36 × 10^−4^)	0.009650 *** (2.18 × 10^−4^)	0.010058 *** (1.67 × 10^−4^)	0.009572 *** (1.71 × 10^−4^)	0.009685 *** (1.66 × 10^−4^)	0.009922 *** (2.87 × 10^−4^)	0.010268 *** (1.22 × 10^−4^)	0.010080 *** (6.36 × 10^−5^)	0.0097288 *** (4.72 × 10^−4^)
*Occu2*	0.002878 *** (3.64 × 10^−5^)	0.003376 *** (6.99 × 10^−5^)	0.003430 *** (9.55 × 10^−5^)	0.003463 *** (1.24 × 10^−4^)	0.003485 *** (1.23 × 10^−4^)	0.004005 *** (9.75 × 10^−5^)	0.004180 *** (1.56 × 10^−4^)	0.004102 *** (7.27 × 10^−5^)	0.004365 *** (3.36 × 10^−5^)	0.003553 *** (2.77 × 10^−4^)
*Occu3*	0.001010 *** (3.29 × 10^−5^)	0.001080 *** (7.19 × 10^−5^)	0.000964 *** (9.73 × 10^−5^)	0.000758 *** (1.22 × 10^−4^)	0.001263 *** (1.25 × 10^−4^)	0.001208 *** (1.20 × 10^−4^)	0.001262 *** (1.45 × 10^−5^)	0.001095 *** (7.09 × 10^−5^)	0.001167 *** (6.59 × 10^−5^)	0.0010291 *** (2.88 × 10^−4^)
*Occu4*	−0.000572 *** (3.18 × 10^−5^)	−0.000098 (9.31 × 10^−5^)	−0.000007 (1.22 × 10^−4^)	0.000072 (1.42 × 10^−4^)	0.000351 *** (1.07 × 10^−4^)	0.000837 *** (1.54 × 10^−4^)	0.001303 *** (1.58 × 10^−4^)	0.001301 *** (1.15 × 10^−4^)	0.001822 *** (3.86 × 10^−5^)	0.0004284 (2.98 × 10^−4^)
*Occu5*	−0.001535 *** (4.32 × 10^−5^)	−0.000793 *** (7.92 × 10^−5^)	−0.001171 *** (9.15 × 10^−5^)	−0.001043 *** (1.44 × 10^−4^)	−0.001034 *** (1.38 × 10^−4^)	−0.000505 *** (1.08 × 10^−4^)	−0.000123 (1.74 × 10^−4^)	−0.0000037 (9.60 × 10^−4^)	0.001103 *** (4.51 × 10^−5^)	−0.000768 ** (3.14 × 10^−4^)
*Occu6*	−0.002188 *** (5.77 × 10^−5^)	−0.002127 *** (1.15 × 10^−4^)	−0.002965 *** (1.09 × 10^−4^)	−0.003494 *** (1.62 × 10^−4^)	−0.003413 *** (2.843 × 10^−4^)	−0.003480 *** (1.51 × 10^−4^)	−0.003644 *** (2.78 × 10^−4^)	−0.002582 *** (1.48 × 10^−4^)	−0.002820 *** (5.61 × 10^−5^)	−0.002998 *** (4.47 × 10^−4^)
*Occu7*	0.005651 *** (4.37 × 10^−5^)	0.006392 *** (8.0 × 10^−5^)	0.006097 *** (1.21 × 10^−4^)	0.006086 *** (1.19 × 10^−4^)	0.006157 *** (1.60 × 10^−4^)	0.006487 *** (1.05 × 10^−4^)	0.006232 *** (1.69 × 10^−4^)	0.006525 *** (7.84 × 10^−5^)	0.007295 *** (5.27 × 10^−5^)	0.0062191 *** (3.15 × 10^−4^)
*Occu8*	−0.003012 *** (7.05 × 10^−5^)	−0.005679 *** (2.84 × 10^−4^)	−0.002506 *** (7.14 × 10^−4^)	−0.001279 ** (5.67 × 10^−4^)	−0.001911 *** (5.91 × 10^−4^)	−0.002600 *** (3.16 × 10^−4^)	−0.002705 *** (7.67 × 10^−4^)	−0.004767 *** (2.89 × 10^−4^)	−0.005625 *** (3.55 × 10^−4^)	−0.0030964 ** (1.53 × 10^−3^)
*Constant*	1.343386 *** (2.02 × 10^−5^)	1.344819 *** (4.04 × 10^−4^)	1.346452 *** (4.81 × 10^−4^)	1.34677 *** (9.73 × 10^−4^)	1.346595 *** (8.64 × 10^−4^)	1.347518 *** (1.06 × 10^−3^)	1.34864 *** (5.50 × 10^−4^)	1.349212 *** (3.10 × 10^−4^)	1.34861 *** (1.56 × 10^−4^)	1.347459 *** (1.08 × 10^−3^)
Obj. Value	−8.20	−10.91	−10.47	−10.11	−11.61	−9.39	−11.17	−10.68	−11.13	−−
Adj. R^2^	−−	−−	−−	−−	−−	−−	−−	−−	−−	0.5701

Note 1: Standard errors are in parentheses, presented in scientific notation. * Indicates significant at the 10% level; ** indicates significant at the 5% level; *** indicates significant at 1% level. Note 2: In the genqreg STATA code, the generalized quantile regression estimator is estimated via Markov chain Monte Carlo (MCMC) optimization procedure. In this study, MCMC with 10,000 draws and 5000 burn was implemented to obtain robust results.

**Table 4:** The estimated value of statistical life by wage.

Model	Hourly Wage ($/h)	Monthly Wage ($/Month)	VSL (Million $)
UQR: 10%	4.46	822.91	8.91
UQR: 20%	4.86	855.83	7.10
UQR: 30%	5.35	921.66	7.43
UQR: 40%	5.76	987.49	9.17
UQR: 50%	6.17	1086.24	9.74
UQR: 60%	6.86	1168.53	8.86
UQR: 70%	7.77	1316.66	11.78
UQR: 80%	9.21	1579.99	25.46
UQR: 90%	11.32	1974.98	31.90
2SLS	7.25	1254.93	6.05

UQR: unconditional quantile regression model.

**Table 5:** The estimated value of statistical life by wage and age (million $).

Model	VSL 20 Years Old	VSL 30 Years Old	VSL 40 Years Old	VSL 50 Years Old	VSL 60 Years Old
UQR: 10%	4.54	6.81	9.08	11.35	13.62
UQR: 20%	3.62	5.43	7.24	9.05	10.87
UQR: 30%	3.79	5.68	7.58	9.47	11.37
UQR: 40%	4.67	7.01	9.35	11.68	14.02
UQR: 50%	4.96	7.45	9.93	12.41	14.89
UQR: 60%	4.52	6.78	9.04	11.30	13.56
UQR: 70%	6.01	9.01	12.02	15.02	18.02
UQR: 80%	12.98	19.47	25.96	32.45	38.94
UQR: 90%	16.26	24.40	32.53	40.66	48.79
2SLS	3.09	4.63	6.17	7.71	9.26

## References

[1] United States Environmental Protection Agency (2016). Valuing Mortality Risk Reductions for Policy: A Meta-Analytic Approach. https://yosemite.epa.gov/sab/sabproduct.nsf/0/0CA9E925C9A702F285257F380050C842/$File/VSL%20white%20paper_final_020516.pdf.

[2] Viscusi W.K., Aldy J.E. (2003). The value of a statistical life: A critical review of market estimates throughout the world. J. Risk Uncertain..

[3] Viscusi W.K., Huber J., Bell J. (2014). Assessing whether there is a cancer premium for the value of a statistical life. Health Econ..

[4] Yang Y., Luo L., Song C., Yin H., Yang J. (2018). Spatiotemporal assessment of PM_2.5_-related economic losses from health impacts during 2014–2016 in China. Int. J. Environ. Res. Public Health.

[5] Furuya S., Chimed-Ochir O., Takahashi K., David A., Takala J. (2018). Global asbestos disaster. Int. J. Environ. Res. Public Health.

[6] Xiao J., Li X., Zhang Z. (2016). DALY-based health risk assessment of construction noise in Beijing, China. Int. J. Environ. Res. Public Health.

[7] Tekesin C., Ara S. (2014). Measuring the value of mortality risk reductions in Turkey. Int. J. Environ. Res. Public Health.

[8] Hammit J.K., Robinson L.A. (2011). The income elasticity of the value per statistic life: Transferring estimates between high and low income populations. J. Benefit Cost Anal..

[9] Hammitt J.K., Graham J.D. (1999). Willingness to pay for health protection: Inadequate sensitivity to probability. J. Risk Uncertain..

[10] Corso P.S., Hammitt J.K., Graham J.D. (2001). Valuing mortality—Risk reduction: Using visual aids to improve validity of contingent valuation. J. Risk Uncertain..

[11] Alberrini A., Cropper M., Krupnick M., Simon N.B. (2004). Does the value of a statistical life vary with age and health status? Evidence from the US and Canada. J. Environ. Econ. Manag..

[12] Hammitt J.K., Haninger K. (2010). Valuing fatal risks to children and adults: Effects of disease, latency, and risk aversion. J. Risk Uncertain..

[13] Cameron T.A., DeShazo J.R. (2010). Euthanizing the value of a statistic life. Rev. Environ. Econ. Policy.

[14] Cameron T.A., DeShazo J.R., Johnson E.H. (2010). The effect of children on adult demands for health—Risk reductions. J. Health Econ..

[15] Chestnut L.G., Rowe R.D., Breffle W.S. (2012). Economic valuation of mortality—Risk reduction: Stated preference estimates from the United States and Canada. Contemp. Econ. Policy.

[16] Cameron T.A., DeShazo J.R. (2013). Demand for health risk reductions: A cross-national comparison between the U.S. and Canada. J. Risk Uncertain..

[17] Yang Z., Liu P., Xu X. (2016). Estimation of social value of statistical life using willingness-to-pay method in Nanjing, China. Accid. Anal. Prev..

[18] Evan M.F., Schaur G. (2010). A quantile estimation approach to identify income and age variation in the value of a statistical life. J. Environ. Econ. Manag..

[19] Kniesner T.J., Viscusi W.K., Ziliak J.P. (2010). Policy relevant heterogeneity in the value of statistical life: New evidence from panel data quantile regressions. J. Risk Uncertain..

[20] Viscusi W.K. (2003). Racial differences in labor market values of statistical life. J. Risk Uncertain..

[21] Smith V.K., Evans M.F., Kim H., Taylor D.H. (2004). Do the near-elderly value mortality risks differently?. Rev. Econ. Stat..

[22] Viscusi W.K. (2004). The value of life: Estimates with risks by occupation and industry. Econ. Inq..

[23] Kniesner T.J., Viscusi W.K. (2005). Value of a statistical life: Relative position vs. relative age. Am. Econ. Rev..

[24] Viscusi W.K., Aldy J.E. (2007). Labor market estimates of the senior discount for the value of statistical life. J. Environ. Econ. Manag..

[25] Aldy J.E., Viscusi W.K. (2008). Adjusting the value of a statistical life for age and cohort effects. Rev. Econ. Stat..

[26] Viscusi W.K., Hersch J. (2008). The mortality cost to smokers. J. Health Econ..

[27] Hersch J.H., Viscusi W.K. (2010). Immigrant status and the value of statistical life. J. Hum. Resour..

[28] Scotton C.R., Taylor L.O. (2011). Valuing risk reductions: Incorporating risk heterogeneity into a revealed preference framework. Resour. Energy Econ..

[29] Scotton C.R. (2013). New risk rates, inter-industry differential and the magnitude of VSL estimates. J. Benefit Cost Anal..

[30] Viscusi W.K., Masterman C.J. (2017). Income elasticities and global values of a statistical life. J. Benefit Cost Anal..

[31] Viscusi W.K. (2011). Policy challenges of the heterogeneity of the value of statistical life. Found. Trends Econom..

[32] Firpo S., Fortin N.M., Lemieux T. (2009). Unconditional quantile regressions. Econometrica.

[33] Borah B.J., Basu A. (2013). Highlighting differences between conditional and unconditional quantile regression approaches through an application to assess medication adherence. Health Econ..

[34] Frölich M., Melly B. (2013). Unconditional quantile treatment effects under endogeneity. J. Bus. Econ. Stat..

[35] Powell D. (2017). Quantile treatment effects in the presence of covariates. Quantile Regression 2017.

[36] Wang W. (1987). Estimation of Value of Statistical Life. Master’s Thesis.

[37] Hsueh L.M., Wang S.W. (1987). Evaluation of Value of Statistical Life for Workers in Taiwan Area: Theory and Practice of Wage Premium of Job-Related Risk.

[38] Liu J.-T., Zhan F.-G. (1989). Estimation of the value of statistical life of workers in Taiwan: Analysis from 1982 to 1986. Acad. Econ. Pap..

[39] Liu J.T., Hammitt J.K., Liou J.L. (1997). Estimated hedonic wage function and value of life in developing country. Econ. Lett..

[40] Liu J.-L. (2011). The Older the Less Value? An Analysis in the Effect of Age on the Value of Life.

[41] Koenker R., Hallock K.F. (2001). Quantile regression. J. Econ. Perspect..

[42] Koenker R. (2005). Quantile Regression.

[43] Kniesner T.J., Viscusi W.K., Woock C., Ziliak J.P. (2007). Pinning Down the Value of Statistical Life.

[44] Mardones C., Riquelme M. (2018). Estimation of the value of statistical life in Chile and extrapolation to other Latin American countries. Lat. Am. Res. Rev..

[45] Moore M.J., Viscusi W.K. (1988). The quantity-adjusted value of life. Econ. Inq..

[46] Directorate-General of Budget, Accounting and Statistic, Executive Yuan (2014). Manpower Utilization Survey.

[47] Occupational Injuries Ratio per Thousandth under Labor Insurance. https://statfy.mol.gov.tw/.

